# Mr. Ring, on the Pustulous Eruptions of Animals

**Published:** 1804-05-01

**Authors:** John Ring

**Affiliations:** New Street, Hanover Square


### *To the Editors of the Medical and Physical Journal.*

GENTLEMEN,

MR. DUNNING, in his Observations on Vaccination, has given an account of an eruptive disease, to which poultry are subject in the East Indies. It is called gooty, which signifies the small-pox. This account was communicated to Mr. Dunning by Dr. Jenner.

Being informed by Capt. Money, that he had seen the disorder, I desired to know some particulars concerning it; and he favoured me with the following statement:

"When I was at Madras in 1794, I purchased twelve dozen of Pondicherry capons, with the intention of preserving them in Bengal for the homeward bound passage. They had not been long in Calcutta, before an eruptive disease made its appearance upon several, and, in the course of a month, extended to and annihilated the whole stock.

"The feathers on the back and breast of many dropped off; and the skin appeared perfectly red. Others died suddenly without loss of feathers; and all died without any diminution of flesh.

"Having related this remarkable instance of fatality, upon my return to the Coast, I was informed that it was not uncommon; that fowls were subject to a disease similar to the small-pox; and that, in some places, they were even inoculated, for the advantage of having it in a milder manner.

This account agrees in most respects with that received by Dr. Jenner from a gentleman who had resided many years at Bengal. That gentleman, however, stated, that inoculation was practised to lessen the evil, but in vain.

Dr. Ivory,

Dr. Ivory, who resided at Bengal twelve years, informs me, that turkeys are subject to a similar disease; which is called by the same name as the small-pox, gooty; and by the Hindoos matah, which is pronounced mawtaw.

Major Magra was told it was the small-pox, and that it always proves fatal. He landed twelve turkies from Sicily, and they all died. It is impossible to breed them at Tunis, although fowls are very plenty there.

I am informed by Major Magra, who resided six years at Tunis, in the character of His Majesty's Agent, and Consul General, that the same disease prevails among turkies on the Coast of Barbary. Their heads swell, and are affected with pustulous eruptions, and they become blind. The disorder is supposed to be infectious.

I have not been able to learn that the chicken-pox of the human species originates in the fowl from which it derives its name. It is probably so denominated only from its resemblance to that affection.

A popular opinion prevails in some parts of England, that the measles originate in swine, and are communicated by the brute animal to the human species. This, we have reason to believe, is a vulgar error; but the history of vaccination proves that popular opinions deserve attention. It is a popular opinion, that the chicken-pox, and the swine-pox, as it is called, in the human species, are two distinct diseases; we have the most respectable medical authority to the contrary; but some cases which I have seen, and others which I have heard, convince me that the subject is worthy of further investigation.

The chicken-pox is sometimes called by another name. Mrs. Wait, No. 6, Husband Street, Carnaby Market, informs me, that when she lived at Berkhamstead, her children had a disorder called the blisters; and that it was contagious. Hence it is probable that it was the chicken-pox, under another denomination.

This disease, however mild, assumes a certain degree of importance, in consequence of the probability of its being mistaken for the small-pox. No error is more common, or more serious than this. I therefore consider it a duty, to point out every mark of discrimination between the two disorders. In addition to others which I have noticed in my treatise on the cow-pox, I shall here mention one, communicated to me by Mr. Hurlock, namely, that the small-pocks have at first a depression at the apex, which the chicken-pocks have not; and hence it is common to hear the expression, the pocks begin to fill.

I can-

I cannot conclude, without recommending a little more attention to that "shadow of a disease," as it has been called, the chicken-pox; since there is no cause from which vaccination has suffered more, or more unjustly, than from this affection being mistaken for the small-pox.

I am, &c.

JOHN RING.

*New Street, Hanover Square, April* 12, 1804.

